# Preliminary study of regionalised centre-of-pressure analysis in patients with juvenile idiopathic arthritis

**DOI:** 10.1186/1757-1146-5-S1-O15

**Published:** 2012-04-10

**Authors:** Gordon J Hendry, Danny Rafferty, Ruth Semple, Janet M Gardner-Medwin, Debbie E Turner, James Woodburn

**Affiliations:** 1 School of Health and Life Sciences, Glasgow Caledonian University, Glasgow, Lanarkshire, G4 0BA, UK; 2 University of Western Sydney, Sydney, NSW, Locked Bag 1797, Australia; 3 University of Glasgow, Glasgow, Lanarkshire, G12 8QQ, UK

## Background

Patients with Juvenile idiopathic arthritis (JIA) may exhibit altered plantar pressure distributions as a result of foot pain and/or deformities [1,2]. Previous work involving adult patients with rheumatoid arthritis suggests that altered loading patterns can be characterised by delayed transfer of the centre-of-pressure to the forefoot [3]. The aim of this study was to compare centre-of-pressure characteristics in pre-defined areas of the foot between patients with JIA and children without JIA.

## Methods

Fourteen patients with JIA (10 female, 4 male) with a mean age of 12.4 years (SD 3.2), and 10 controls (6 female, 4 male) with a mean age of 12.5 years (SD 3.4) were studied. Foot deformity and impairment scores were recorded using the Structural Index (SI) [4] and the Juvenile Arthritis Foot Disability Index (JAFI) [5]. The progression of the centre of pressure through the left foot, heel, midfoot, forefoot and toe regions was measured using an EMED-X pressure platform. Variables analysed were the average and maximum velocity, velocity at 50% foot length, and the duration of the centre of pressure. Mean differences between groups and 95% confidence intervals (CI) were calculated using the *t-*distribution.

## Results

In the JIA group, participants exhibited mild-to-moderate levels of foot impairments, and mild levels of forefoot deformities. No significant differences were observed between group means for all CoP variables (table 1). A trend towards a slower VCoP_ave_ at the midfoot in the patients with JIA was observed.

**Table 1:** Mean (standard deviation) of the regionalised CoP variables for the JIA and able-bodied control groups.

Variable	Region	JIA (n=14)	Controls (n=10)	Mean difference (95% CI)
VCoP_ave_ (m/s)	Foot	0.28 (0.03)	0.31 (0.05)	0.02 (0.00, 0.05)
	Heel	0.27 (0.08)	0.29 (0.10)	0.02 (-0.02, 0.08)
	Midfoot	0.42 (0.10)	0.48 (0.08)	0.06 (0.00, 0.12)
	Forefoot	0.22 (0.03)	0.23 (0.05)	0.01 (-0.02, 0.03)
	Toes	0.76 (0.48)	0.69 (0.25)	-0.06 (-0.40, 0.28)

VCoP_max_ (m/s)	Foot	1.39 (0.77)	1.38 (0.60)	-0.01 (-0.62, 0.60)
	Heel	0.59 (0.14)	0.65 (0.17)	0.06 (-0.07, 0.19)
	Midfoot	0.63 (0.32)	0.66 (0.09)	0.03 (-0.18, 0.25)
	Forefoot	0.83 (0.42)	0.86 (0.33)	0.03 (-0.29, 0.36)
	Toes	1.23 (0.73)	1.31 (0.57)	0.08 (-0.49, 0.66)

DCoP (% stance)	Heel	25.83 (6.96)	26.01 (6.91)	0.18 (-5.78, 6.14)
	Midfoot	20.71 (4.14)	19.19 (3.63)	-1.52 (-4.89, 1.87)
	Forefoot	46.54 (8.92)	48.20 (7.51)	1.66 (-5.53, 8.85)
	Toes	6.92 (3.27)	6.60 (1.22)	-0.32 (-2.58, 1.94)

VCoP (m/s)	50% foot length	46.54 (7.28)	45.21 (7.93)	-1.34 (-7.82, 5.15)

VCoP_ave_ (m/s): average velocity of the centre of pressure; VCoP_max_ (m/s): maximum velocity of the centre of pressure; DCoP (% stance): duration of the centre of pressure; CI: confidence interval

## Conclusions

Foot impairment and deformity scores may represent residual disease impairments that do not appear to profoundly affect foot function. The low levels of forefoot deformity in the JIA group may explain the lack of compensatory off-loading strategies observed. Sub-classification of patients by locality of symptoms in future studies may be useful to determine the clinical utility of centre-of-pressure analysis in this patient population.
